# Characterization of human skeletal stem cells in closed and open tibia fractures: a single center pilot study

**DOI:** 10.3389/fphys.2025.1638064

**Published:** 2025-10-01

**Authors:** Rubén Tresgallo-Parés, David Morales, Shannon Tse, Aziz Saade, Ellen Fitzpatrick, Sean T. Campbell, Gillian L. Soles, Thomas H. Ambrosi, Mark A. Lee, Augustine M. Saiz

**Affiliations:** University of California Davis Department of Orthopaedic Surgery, Sacramento, CA, United States

**Keywords:** human skeletal stem cells, tibia fracture, open fracture, osteogeneic differentiation, colony forming assay, orthopaedic trauma

## Abstract

**Introduction:**

Tibial shaft fractures are the most common long bone fractures and carry a significant risk of nonunion, particularly in open injuries. This increased risk has been attributed to heightened activation of damage-associated molecular pathways, cellular senescence, and alterations in the fracture milieu. However, most supporting evidence arises from animal studies under controlled conditions, and the cellular and biochemical environment of human traumatic fractures remains poorly understood. While mechanical and patient-related factors have been associated with impaired healing, the biological mechanisms are not well defined. Human skeletal stem cells (hSSCs) play a critical role in bone regeneration and may provide insight into differential healing responses. This study aimed to characterize hSSC recruitment and functional capacity in open versus closed tibial fractures.

**Methods:**

A prospective pilot study of patients undergoing acute tibial shaft fixation at a Level 1 trauma center was conducted. During intramedullary nailing, reamings were collected for analysis. hSSCs were isolated via flow cytometry. Functional assays included colony-forming unit (CFU) counts and *in vitro* osteogenic differentiation via Alizarin Red staining. CD146^+^ osteostromal cells were quantified, and serum alkaline phosphatase (ALP), IL-6, and HbA1c levels were analyzed. Statistical comparisons were made between groups, and outliers were identified using the ROUT method (Q = 5%).

**Results:**

19 patients with isolated tibial shaft fractures (8 closed, 11 open) treated with intramedullary nailing were included. hSSC frequency was significantly lower in open fractures compared to closed fractures after outlier exclusion (2.75% ± 1.67% vs. 5.64% ± 5.80, p = 0.032), suggesting reduced early recruitment. However, no significant differences were observed in CFU capacity (0.0078 ± 0.0071 vs. 0.0156 ± 0.0117, p = 0.221) or osteogenic differentiation (1.24 ± 0.22 vs. 1.52 ± 0.85, p = 0.419). CD146^+^ cell levels and serum markers were similar between groups. ALP levels correlated strongly with CD146^+^ cell abundance in closed fractures (*ρ* = 0.80, p = 0.02) but not with hSSC levels.

**Conclusion:**

Open tibial fractures demonstrate reduced early hSSC recruitment compared to closed fractures in the acute period of fracture healing, while *in vitro* stem cell function appears preserved. These findings support a model in which impaired healing in open fractures may result from reduced hSSC recruitment rather than dysfunction. Larger studies with long-term clinical follow-up are warranted to validate these results and explore therapeutic strategies targeting the hSSC niche to enhance fracture healing in high-risk populations.

## Introduction

Tibial shaft fractures are the most common long bone fractures, with an estimated incidence ranging from two to ten per 1,000 to 10,000 people annually in the United States. Tibia fractures remain prone to complications, particularly nonunion, which occurs in approximately 5%–15% of cases (Audigé et al., 2005; Mills et al., 2017), which can lead to chronic pain, prolonged functional, psychological, and physical burdens on patients, and increased healthcare costs (Johnson et al., 2019; Brinker et al., 2013; Antonova et al., 2013). Open tibia fractures carry more than twice the risk of nonunion compared to closed injuries (Fong et al., 2013). While certain injury characteristics, patient comorbidities, and technical surgical factors contribute to this risk, the underlying biological mechanisms driving nonunion are not fully understood. Emerging evidence suggests that open fractures may enhance activation of damage-associated molecular pathways, cellular senescence, and changes in the local molecular environment that impair healing. However, most of these insights stem from controlled from animal models (Lounev et al., 2009; Chan et al., 2015), and the human fracture milieu particularly in the early postoperative period remains poorly characterized.

Recently, attention has been directed toward the cellular environment of fracture tissue, particularly the role of human skeletal stem cells (hSSCs) in fracture healing (Chan et al., 2018; Ambrosi et al., 2021a; Goodnough et al., 2020; Ambrosi and Chan, 2021; Xu et al., 2020). hSSCs, which sit at the apex of the skeletal lineage tree, are capable of self-renewal and multilineage differentiation into bone, cartilage, and stroma (Chan et al., 2018; Ambrosi et al., 2020). Unlike CD146^+^ stromal populations, which are heterogeneous and prone to culture-induced artifacts, hSSCs can be precisely isolated via fluorescence-activated cell sorting (FACS) using a well-defined surface marker profile—PDPN^+^CD146^−^CD73^+^CD164^+^—first identified by Chan et al. as defining a clonogenic, multipotent population capable of generating bone, cartilage, and stromal progenitors (Chan et al., 2018; Ambrosi et al., 2021a; Ambrosi and Chan, 2021). This phenotypic clarity allows for accurate functional and molecular characterization. Chan et al. have further clarified hSSC identity and downstream progeny in both mouse and human models, enabling deeper investigation into their role in skeletal regeneration (Chan et al., 2015; Chan et al., 2018). A case study by Goodnough et al. demonstrated that hSSCs isolated at the time of surgery from a patient who later developed nonunion exhibited impaired osteogenic differentiation *in vitro*, while those from a patient who went on to heal normally retained robust activity, suggesting that hSSCs have a role in fracture healing (Goodnough et al., 2020).

Although certain injury patterns and surgical factors are known to influence nonunion rates (Bhandari et al., 2001), predicting individual healing capacity remains a challenge in orthopaedic trauma. Characterization of hSSCs offer a promising approach to assessing a patient’s biologic healing potential. hSSCs can be prospectively isolated from fracture hematoma at the time of fracture fixation using FACS, as described by Ambrosi et al. (2021a) (Ambrosi and Chan, 2021). This study builds on that work by examining the cellular composition and molecular environment of fracture sites in open versus closed tibial shaft fractures, with specific emphasis on hSSC presence and function. By correlating these findings with clinical outcomes, we aim to determine whether early hSSC dysfunction or deficiency is associated with subsequent nonunion in human fracture healing.

This study aims to characterize the presence, viability, and osteogenic activity of hSSCs in open versus closed tibial shaft fractures. We hypothesize that open fractures will demonstrate significantly impaired hSSC function compared to closed injuries. By identifying key differences in the cellular composition of healing environments, this work may provide foundational insights into the mechanisms of nonunion and suggest novel therapeutic targets to enhance skeletal repair.

## Materials and methods

### Patients and clinical data

Following approval by the local Institutional Review Board approval, a prospective pilot study was initiated in January 2024 at a single academic level 1 trauma center. Adult patients aged 18–65 years with isolated open or closed tibia shaft fractures treated with intramedullary nailing were eligible for inclusion. Exclusion criteria included evidence of active infection at the fracture site, ipsilateral lower extremity injuries, prior surgery or trauma involving the same tibia, pregnancy, incarceration, and non-English speaking patients (due to limitations in consenting).

Patient age, sex, body mass index (BMI), history of smoking, diabetes, and American Society of Anesthesiologists (ASA) score was collected. Open fracture status, Gustilo Anderson classification and time from emergency department arrival to IMN procedure start was also recorded. Postoperative outcomes included duration of follow-up, nonunion, deep infection requiring reoperation, superficial infection managed nonoperatively, and any unplanned revision surgery.

### Sample collection

All procedures were performed by a fellowship-trained orthopaedic trauma surgeon. Samples were obtained intraoperatively at the time of intramedullary nailing in the form of reamings collected from the tibia as they were extruded from the reamer and intramedullary canal. This procedure was performed without the need for additional incisions or deviation from standard operative technique. All samples were processed on the same day of collection in a central laboratory. Peripheral blood samples were also collected perioperatively via venipuncture to assess hemoglobin A1c (HbA1c), alkaline phosphatase (ALP) and interleukin-6 (IL6) serum levels.

### Flow cytometric isolation of skeletal progenitor cells

**FIGURE 1:**
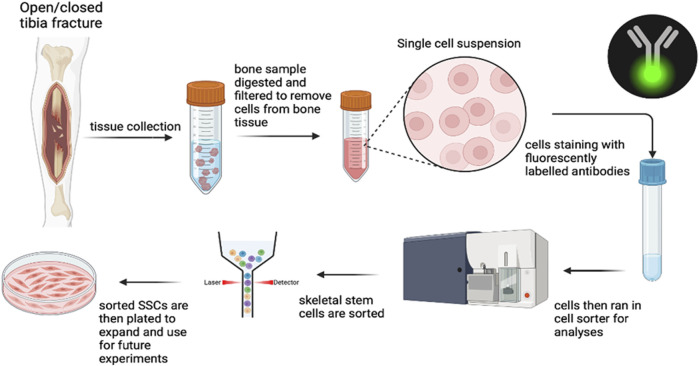
Methodology of flow cytometric isolation of hSSCs.

hSSCs were isolated from fracture hematoma via flow cytometry using cell surface marker profiles (Chan et al., 2018; Hoover et al., 2023). Samples were washed in ice-cold phosphate-buffered saline (PBS), minced, and digested in M199 (Thermo Fisher Scientific, cat#11150067) containing 2.2 mg/mL type II collagenase (SigmaAldrich, cat#C6885) and 100 U/mL DNase I (Worthington, cat#NC9199796) for 60 min at 37 °C under constant agitation. The digest was strained through a 100 µm nylon mesh, quenched with staining medium (2% FBS, 1% Penicillin–Streptomycin, 0.1% Poloxamer 188 in PBS), and pelleted at 200 g for 5 min at 4 °C. Erythrocytes were removed using ACK lysis buffer (Thermo Fisher Scientific, cat#A1049201), followed by additional washing and pelleting. Figure 1 demonstrates the methodology of flow cytometric isolation of hSSCs.

Cells were incubated with fluorochrome-conjugated antibodies targeting CD45, CD235a, CD31, CD202b/Tie2, streptavidin APC-AlexaFlour750, CD146, PDPN, CD164 and CD73. Viability was assessed by DAPI staining. Cell sorting was performed on a Cytek Aurora CS (Cytek) with a 100 µm nozzle. Gating strategies were determined using fluorescence-minus-one controls, and post-sort purity and analysis were carried out in FlowJo v10.10. CD146^+^ osteostromal cell populations were quantified separately for correlation with biochemical markers.

### hSSC culture and differentiation

Freshly sorted primary hSSCs were cultured in αMEM (Thermo Fisher Scientific, cat#12561056) supplemented with 10% human platelet-derived lysate (HPL; Stem Cell Technologies, cat#06962), 1% penicillin–streptomycin and 0.01% heparin. Cultures were maintained at 37 °C in a humidified 5% CO_2_ incubator, with medium changes every 2–3 days.

**FIGURE 2:**
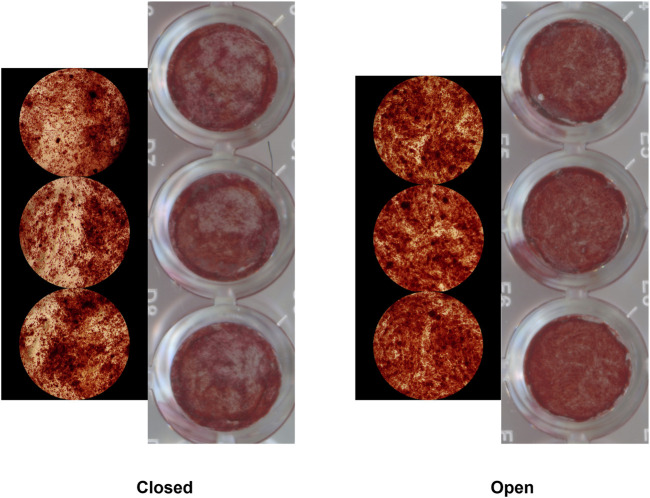
Alizarin Red Staining of hSSCs isolated from patients with closed (left) and open (right) tibia fractures following osteogenic differentiation.

For colony-forming unit (CFU) assays, hSSCs were seeded at clonal density and incubated for 10–14 days. Colonies containing ≥50 cells were counted manually under light microscopy. For osteogenic differentiation, at 80–90% confluence, cultures were switched to osteogenic induction medium containing αMEM, 10% FBS, 1% penicillin–streptomycin, 100 nM dexamethasone (MP Biomedicals, cat#194561), 10 mM β-glycerophosphate (SigmaAldrich, cat#G9891), and 2.5 mM ascorbic acid 2-phosphate (SigmaAldrich, cat#A8960). Medium was refreshed every other day. On day 10, cells were fixed in 4% paraformaldehyde, stained with 2% Alizarin Red S (ARS) (SigmaAldrich, cat#A5533), and excess dye was removed with PBS. Bound stain was solubilized in 20% methanol/10% acetic acid solution for 30 min and quantified at 450 nm using a microplate reader (Figure 2). ARS absorbance values were used to quantify osteogenic differentiation. All differentiation assays were performed in triplicate for each patient sample.

### Statistical analysis

Patients were categorized into two groups based on their open fracture status (open or closed). The results of laboratory outcomes and serum blood results were correlated with clinical outcomes. Appropriate tests of significance were used to compare data from both groups, including t-tests for continuous variables and chi-squared and fisher’s exact tests for categorical variables. Spearman rank correlation was used to assess associations between ALP, a marker for osteogenesis, and CD146^+^ or hSSC counts. Outliers were identified using the Robust Regression and Outlier Removal (ROUT) method with a false discovery rate (Q) of 5%. Outlier detection was performed on a variable-specific basis; values identified as outliers were excluded only from analyses involving that specific variable, while all other data from the patient were retained for related analyses. Sensitivity analyses were conducted with and without outliers to assess the robustness of findings. Any exclusions and their impact on statistical significance are reported. All statistical analyses were performed using Prism (GraphPad Software, Boston MA) and a p value of <0.05 was considered to be statistically significant.

## Results

A total of 22 patients were initially enrolled in the study. Three were excluded: two due to incomplete consent documentation and one due to loss of laboratory data. Nineteen patients were included in the final analysis. The cohort consisted of 6 females and 13 males, with a mean age of 40.5 ± 22.1 years and a mean BMI of 30.4 ± 7.3. There were 8 closed and 11 open tibial shaft fractures. No significant demographic differences were observed between groups (Table 1). Of the open fractures, there was one Gustilo-Anderson type I, five type II, two type IIIA, and three type IIIB fractures. There were no differences between the closed and open fracture groups with regards to HbA1c, ALP, and IL6 levels (Table 2). The mean time from emergency department admission to the IMN procedure start was 74.4 h in the closed fracture group and 101.8 h in the open fracture group (p = 0.700).

**TABLE 1:** Patient demographics of closed and open fracture groups.

Variable	Closed fracture	Open fracture	p value
Patients, n (%)	8 (42.1%)	11 (57.9%)	
Age, mean ± SD	36.0 ± 15.6	43.8 ± 26.0	0.462
Male sex, n (%)	4 (50.0%)	9 (81.8%)	0.319
BMI, mean ± SD	27.9 ± 6.4	32.2 ± 7.6	0.214
Diabetes, n (%)	0 (0%)	2 (18.2%)	0.485
Smoking, n (%)	5 (62.5%)	6 (54.5%)	0.375
ASA, n (%)			
1	2 (25.0%)	1 (9.1%)	0.553
2	3 (37.5%)	5 (45.5%)	
3	2 (25.0%)	5 (45.5%)	
4	1 (12.5%)	0 (0%)	

BMI, body mass index; ASA, american society of anesthesiologists score.

**TABLE 2:** HbA1c, ALP, and IL6 levels of closed and open fracture groups.

Variable	Closed fracture	Open fracture	p value
HbA1c, mean ± SD	5.3 ± 0.4	5.6 ± 0.5	0.175
ALP, mean ± SD	93.5 ± 43.3	69.7 ± 17.0	0.114
IL6, mean ± SD	9.6 ± 8.9	55.2 ± 87.4	0.374

HbA1c, hemoglobin A1c; ALP, alkaline phosphatase; IL6, interleukin-6.

When analyzing the full dataset including outliers, mean hSSC frequency in closed fractures was 2.57% ± 3.02, compared to 3.94% ± 3.46 in open fractures (p = 0.384). CFU/cell number was 0.0165 ± 0.0139 for closed fractures and 0.0426 ± 0.0739 for open fractures (p = 0.508). CD146^+^ cell percentages were 4.75% ± 6.47 for closed fractures and 4.58% ± 9.25 for open fractures (p = 0.963). Osteogenic differentiation capacity, as determined by ARS staining absorbance, was 1.52 ± 0.85 in closed fractures versus 1.24 ± 0.22 in open fractures (p = 0.419).

**FIGURE 3:**
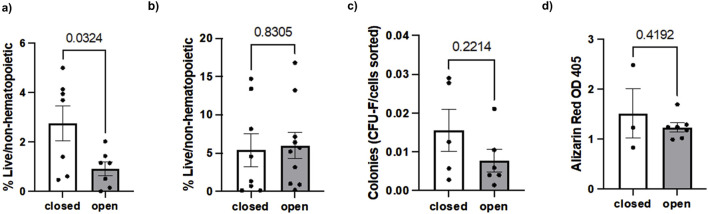
Comparison of **(a)** hSSCs, **(b)** CD146+ osteostromal cells, **(c)** CFU and **(d)** osteogenic differentiation capacity in closed versus open fractures.

Inspection of the dataset revealed potential extreme values in SSC frequency, CFU counts, and CD146^+^ cell percentages. Outliers were therefore excluded using the ROUT method (Q = 5%). Following exclusion, hSSC frequency was significantly higher in closed fractures compared to open fractures (5.64% ± 5.80 vs. 2.75% ± 1.67, p = 0.032). The CFU assay revealed no statistically significant difference in colony-forming ability between closed fractures and open fractures (0.0156 ± 0.0117 vs. 0.0078 ± 0.0071 CFU/cell number, p = 0.221). CD146^+^ cell percentages were 5.39% ± 5.80 for closed fractures and 5.97% ± 5.10 for open fractures (p = 0.831). These results are graphically displayed in Figure 3.

**FIGURE 4:**
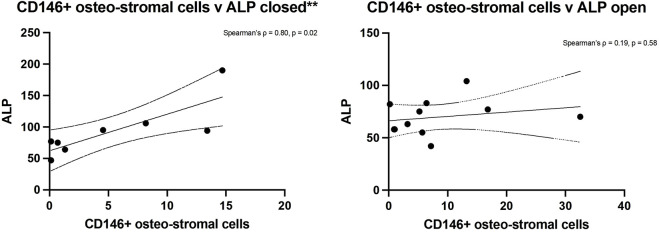
Spearman’s rank analysis between ALP levels and CD146^+^ cells numbers in closed (left) and open (right) fractures.

**FIGURE 5:**
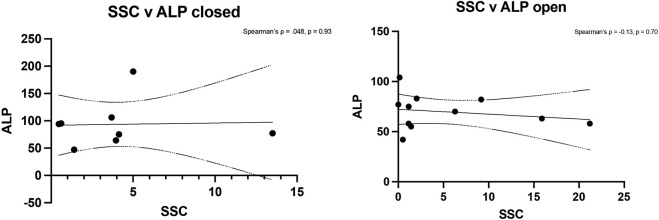
Spearman’s rank analysis between ALP levels and hSSCs in closed (left) and open (right) fractures.

Spearman’s rank analysis showed a strong positive correlation between ALP levels and CD146^+^ cell numbers in closed fractures (Spearman’s ρ = 0.80, p = 0.02) but not in open fractures (ρ = 0.19, p = 0.58) (Figure 4). No significant correlation was observed between ALP and hSSC frequency in either setting (closed: ρ = 0.05, p = 0.93; open: ρ = −0.13, p = 0.70), consistent with the understanding that ALP is predominantly expressed by more committed osteochondral progenitors within the CD146^+^ compartment (Figure 5).

The mean follow-up duration was 167.1 ± 95.1 days. One patient was followed at an outside institution and was excluded from clinical outcome analysis. There were no deep or superficial infections reported in any of the patients. Unplanned revision surgery occurred in 1 patient (12.5%) with a closed fracture who underwent removal of symptomatic hardware, and 3 patients (30.0%) with open fractures (p = 0.588) - one patient had necrotic soft tissue requiring multiple debridements subsequently requiring an amputation, one developed soft tissue ischemia requiring debridement and split thickness skin grafting, and one underwent removal of symptomatic hardware. Of the 12 patients (63.2%) with at least 3 months of follow-up, there were no occurrences of nonunion.

## Discussion

In this prospective pilot study, we investigated the frequency and activity of human skeletal stem cells in fracture tissues from patients with open and closed tibial shaft fractures. Our goal was to evaluate whether differences in hSSC presence or activity might contribute to the disparity in healing outcomes, particularly the increased risk of nonunion associated with open fractures. To our knowledge, this is the first study to directly compare hSSC characteristics in human open versus closed tibial fractures.

We found that hSSC frequency was significantly lower in open fractures compared to closed fractures, suggesting that open injuries are associated with reduced early recruitment of skeletal stem cells at the fracture site. This supports the hypothesis that a diminished cellular reservoir for bone regeneration may contribute to impaired healing in open fractures. Importantly, while hSSC abundance was lower, their intrinsic functionality measured by colony-forming capacity and osteogenic differentiation potential was not significantly different between groups. These findings imply that although open fractures may be biologically disadvantaged at the outset, the regenerative capacity of available hSSCs remains intact.

Additionally, correlation analysis revealed that serum ALP levels were closely aligned with the abundance of CD146^+^ osteostromal progenitors in closed fractures, but showed no meaningful association with the hSSC population in either fracture type. These findings suggest that enzymatic markers of early bone formation may more accurately reflect activity of committed stromal cells, rather than primitive skeletal stem cells, during early healing. Collectively, our data underscore SSC quantity, rather than intrinsic quality, as a key biologic distinction between closed and open tibial fractures.

These observations align with an expanding body of literature emphasizing the central role of hSSCs in skeletal repair. Chan et al. first identified a clonogenic, multipotent hSSC population capable of generating bone, cartilage, and stromal progenitors, but notably lacking adipogenic potential (Chan et al., 2018). These cells, prospectively isolated from fetal and adult bone expand locally in response to acute skeletal injury and possess the ability to generate bone, cartilage, and stromal progenitors—though notably lacking adipogenic potential. Subsequent studies by Ambrosi et al. refined techniques for prospectively isolating site-specific hSSCs using FACS, enabling direct interrogation of their lineage and regenerative potential (Ambrosi et al., 2021a; Ambrosi and Chan, 2021; Hoover et al., 2023). These advances laid the groundwork for investigating how local stem cell environments may influence healing outcomes.

Although we observed no significant differences in CFU counts or osteogenic differentiation capacity between open and closed fractures, this does not diminish the clinical relevance of stem cell abundance. Our results suggest that while hSSC frequency is reduced in open fractures, the intrinsic capacity of these cells to proliferate and differentiate osteogenically is preserved *in vitro*. The clonogenic potential, as assessed by CFU assays, and osteogenic activity, were comparable between groups, indicating that the functional quality of hSSCs remains intact despite differences in their initial recruitment. These findings highlight a key distinction between stem cell quantity and quality—suggesting that a reduced cellular reservoir in open fractures may still be competent for regeneration, but potentially insufficient in number to meet the demands of healing. However, it is important to note that *in vitro* assays may not fully reflect the complexity of the *in vivo* fracture environment, where inflammatory mediators, vascularity, and mechanical stress could significantly alter hSSC behavior and impair healing outcomes.

Previous studies further underscore the clinical relevance of hSSC function in bone healing. Goodnough et al. isolated hSSCs from fracture hematoma in two young patients undergoing ORIF for bilateral forearm fractures, revealing striking functional differences in hSSCs between the patient who healed successfully and the one who developed nonunion. Specifically, hSSCs from the nonunion case lacked osteogenic differentiation capacity despite otherwise similar patient profiles (Goodnough et al., 2020). Similarly, aging has also been shown to impair hSSC activity. Geriatric-derived hSSCs retain clonogenicity but exhibit reduced osteochondral potential, with transcriptomic profiling revealing downregulation of skeletogenic pathways and upregulation of senescence markers (Ambrosi et al., 2020). Ambrosi et al. found that aging murine skeletal stem cells acquire a cell-intrinsic, pro-inflammatory phenotype through interaction with the hematopoietic system, limiting their regenerative potential (Ambrosi et al., 2021b). Extrinsic cues, such as signals from the muscle secretome (Saiz et al., 2019) and exposure to NSAIDs (Goodnough et al., 2022), have also been shown to modulate hSSC behavior.

Recent *in vivo* models demonstrate that endogenous SSCs can be recruited or activated to enhance bone repair. For instance, 3D-printed hydrogel grafts containing neurotrophic factors significantly expanded local SSCs and promoted calvarial bone regeneration in a mouse model (Zhang et al., 2022). In aging models, impaired skeletal healing was reversed by localized delivery of BMP2 in combination with a CSF1 antagonist, which reactivated resident SSCs and improved fracture outcomes (Ambrosi et al., 2021b). Additionally, biomaterial scaffolds functionalized to release chemotactic cues such as SDF-1 have been shown to enhance host progenitor recruitment and osteogenesis without the need for cell transplantation (Pacelli et al., 2017). These studies collectively support the concept that modulating the skeletal niche to enhance endogenous SSC recruitment is a viable therapeutic strategy, with clear translational potential for improving bone regeneration in patients, particularly through scaffold-based or pharmacologic approaches that could be readily adapted for clinical use.

Taken together, our findings highlight a potentially important role for hSSC abundance in fracture healing. The reduced presence of hSSCs in open fractures may help explain the higher rates of nonunion seen in clinical practice. While intrinsic stem cell function appears preserved *in vitro*, it remains unknown how the *in vivo* inflammatory environment, patient age, or pharmacologic exposures may influence hSSC-mediated repair. In our small cohort, no cases of nonunion were observed, likely due to the small sample size and limited follow-up period, which precluded direct clinical correlation between hSSC characteristics and healing outcomes. Nevertheless, these early biologic findings suggest a cellular mechanism that may contribute to impaired healing in more severe fracture patterns. These insights provide a rationale for further investigation into whether modulating the local hSSC environment, such as through biologics, cell therapies, or pharmacologic agents, could improve outcomes in high-risk fractures.

This study has several limitations. As a small, single-center pilot study, our findings may not be generalizable to broader populations. Although open fractures are known to be associated with higher nonunion rates, we observed no nonunions in our cohort, likely due to limited follow-up duration and a small cohort, preventing direct clinical correlation with healing outcomes. We were also unable to control for all patient-specific variables, such as comorbidities and injury severity, which may have influenced hSSC behavior. We did not analyze potential correlations between hSSC frequency and gender or age, as the sample size was too small to detect meaningful differences. The low number of reoperations in our cohort also limited our ability to assess any correlations between reoperation rates and hSSC frequency. Despite these limitations, this study provides foundational insight into the cellular biology of human fracture healing and supports future, larger-scale studies examining hSSCs as both biomarkers and therapeutic targets in orthopaedic trauma care.

## Conclusion

This pilot study demonstrates that human skeletal stem cells are significantly less abundant in open tibial fractures compared to closed fractures, suggesting a potential cellular basis for the higher nonunion rates observed in open injuries. Despite this difference in stem cell recruitment, hSSC functional activity and osteogenic differentiation capacity were preserved *in vitro*, indicating that healing impairment may be more closely related to stem cell depletion rather than dysfunction. These findings highlight the importance of characterizing the cellular composition of human fracture sites and lay the foundation for future investigations into hSSC-targeted diagnostics and regenerative therapies. Larger, longitudinal studies that incorporate clinical outcomes and further in-depth analysis of the obtained cells such as RNA sequencing will be essential to validate these preliminary findings and inform the development of biologically based strategies to enhance fracture healing in high-risk populations.

## Data Availability

The raw data supporting the conclusions of this article will be made available by the authors, without undue reservation.
